# Salinity Stress in Rice: Multilayered Approaches for Sustainable Tolerance

**DOI:** 10.3390/ijms26136025

**Published:** 2025-06-23

**Authors:** Muhammad Ahmad Saleem, Ahmad Khan, Jinji Tu, Wenkang Huang, Ying Liu, Naijie Feng, Dianfeng Zheng, Yingbin Xue

**Affiliations:** 1 National Center of Technology Innovation for Saline-Alkali Tolerant Rice/College of Coastal Agricultural Sciences, Guangdong Ocean University, Zhanjiang 524088, China; ahmadagron01@gmail.com (M.A.S.);; 2 Department of Agronomy, Faculty of Crop Production Sciences, The University of Agriculture, Peshawar 25130, Pakistan; khanahmad@aup.edu.pk

**Keywords:** rice (*Oryza sativa*), soil salinity, salt stress responses, organic amendments, physiological mechanism, molecular mechanism, metabolic pathways

## Abstract

Salt accumulation in arable lands causes significant abiotic stress, resulting in a 10% loss in global arable land area and jeopardizing food production and agricultural sustainability. In order to attain high and sustainable food production, it is imperative to enhance traditional agricultural practices with modern technology to enable the restoration of arable lands afflicted by salinity. This review consolidates recent rice-specific advancements aimed at enhancing salt stress resilience through integrated strategies. We explore the functions of primary and secondary metabolic pathways, organic amendments, microbial symbiosis, and plant growth regulators in reducing the negative impacts of salt. Furthermore, we highlight the significance of emerging genetic and epigenetic technologies, including gene editing and transcriptional regulation, in developing salt-tolerant rice cultivars. Physiological studies reveal salt stress responses in rice plants, biochemical analyses identify stress-related metabolites, microbial investigations uncover beneficial plant–microbe interactions, and molecular approaches enable the identification of key genes—together providing essential insights for developing salt-tolerant rice varieties. We present a comprehensive overview of the multilayered strategies—ranging from agronomic management and physiological adaptations to molecular breeding and microbial applications—that have been developed and refined over recent decades. These approaches have significantly contributed to understanding and improving salinity tolerance mechanisms in rice. This review provides a foundational framework for future research and practical implementation in stress-resilient rice farming systems.

## 1. Introduction

Soil salinity occurs when salts build up in the soil due to natural processes or human activities. Natural causes include high evaporation, low rainfall, rising temperatures, saline groundwater moving upward, salt deposits from oceans, and mineral accumulation [1,2]. These factors are common in arid or semi-arid regions, where “primary salinization” naturally occurs. In contrast, “secondary salinization” (human-caused) is linked to poor farming practices, such as over-irrigation, improper fertilizer use, or inadequate drainage, often seen in humid or sub-humid areas [3]. Salinity harms the physio-chemical and biological characteristics of soil, preventing plant growth and development, soil fertility, and global economic growth [4,5]. Over 800 million ha of land are thought to be saline worldwide, with this amount growing by roughly 1% to 2% per year [6]. An estimated USD 27.2 billion is lost annually in irrigated agriculture through crop loss due to salt-induced soil deterioration [7]. Over 900 million ha of agricultural soil globally were saline [8].

Soil salinity causes a substantial decrease in rice yield. One study found that rice yield decreases by about 29.29% in saline soils compared to non-saline soils [9]. Another report showed average rice production dropping from 4232 kg/ha in low-saline areas to 2663 kg ha^−1^ in highly saline areas [10].

In this review, we systematically summarized the current advancements in strategies for enhancing and sustaining plant productivity in saline soil environments. We provide a thorough examination of cutting-edge strategies, including genetic engineering, altering metabolic pathways, modifying antioxidant mechanisms, applying organic amendments, and using arbuscular mycorrhizal fungus (AMF). We also explored the response of plants to salinity at various levels—from their physical growth to internal chemical processes and genetic activity. By understanding these reactions, we can develop practical solutions to protect crops from salt damage and maintain healthy yields. This study attempts to lay the groundwork for future research centered on creating sustainable solutions for enhancing crop resilience and production in salty environments by incorporating recent research findings. In order to escape persistent salinity, plants have developed numerous adaptation mechanisms. These mechanisms include enhancing the expression of ion transporter or ion pump genes to regulate ion balance; activating the antioxidant enzyme system to remove superoxide anions, etc.; improving metabolic pathways to promote the synthesis of osmoregulation; and establishing symbiosis with mycorrhizal fungi, etc. In addition, researchers counteract crop salt stress by creating salt-tolerant cultivars using genetic engineering and traditional breeding. To control salt, they also use agronomic techniques, including better drainage, irrigation, and soil additives. Furthermore, the utilization of growth regulators, osmoprotectants, and beneficial microorganisms contributes to the improvement of plant resistance, which enhances soil quality and crop yield [11]. Researchers have developed and adopted multiple layered strategies to reduce or overcome salt stress in crops, including rice. These approaches integrate biological, genetic, agronomic, and soil management innovations to enhance plant tolerance and sustain crop productivity under saline conditions.

## 2. Impact of Salinity on Soil Characteristics and Productivity

Salt affects soil structural stability. Tang et al. [12] discovered that the structural properties of silty clay and silt loam were affected by salinity. The percentage of water-stable macroaggregates (0.25–2 mm) in both soil types rose in tandem with the soil salt concentration (SSC). SSC first raised the plant-available water capacity and retention capacity in silt loam to around 14.5 g kg^−1^, after which it started to decline. Over time, the accumulation of sodium degrades soil structure, leading to compaction, lower organic matter content, and poor aeration, which further hinders root growth and water infiltration.

Salt stress also alters the pH of soil. A rise in soil pH consequently increases the OH-ion concentration. However, due to their greater solubility, soil having sodium carbonate (Na_2_CO_3_) had a pH greater than 8.5 or even more than 10, soils containing calcium carbonate (CaCO_3_) and magnesium carbonate (MgCO_3_) have poor solubility due to restricted hydrolysis, resulting in a pH of no more than 8.5 [13,14]. High salinity causes osmotic stress because it raises the number of soluble salts in the soil. Even when water is present, plants find it more difficult to absorb it because of the poor soil water potential caused by this high salt content [15].

Sodium, calcium, and magnesium are the most common cations in saline soil, as are the anions Cl^−^, sulfate (SO_4_^2−^), and bicarbonate (HCO_3_^−^); Na^+^ and Cl^−^ predominate in these soils. Moreover, salt stress results in nutritional imbalances [16]. Salinity affects nutrient dynamics by interfering with nutrient absorption and exchange processes, leading to poor soil structure and reduced soil fertility [17]. Excessive rhizosphere Na^+^ and Cl^−^ levels may prevent essential soil elements from being absorbed by fixation, adsorption, and transformation processes [18].

Salinity decreases water infiltration rates and hydraulic conductivity, particularly in red soil. This results from the buildup of Na^+^ and Cl^−^, which alter soil chemistry and structure, leading to reduced water movement [19]. High salt concentration in soil can lead to clay dispersion and swelling, reducing soil porosity and structural stability. However, the presence of K^+^ relative to Na^+^ can improve soil structural stability [20]. Salinity can reduce the effectiveness of organic matter in retaining water [21]. Gonçalo Filho et al. [22] and Xie et al. [23] emphasized that increased salinity and sodium adsorption ratio (SAR) weaken soil aggregates and decrease organic matter. High levels of Na⁺ promote soil dispersion, whereas Ca^2^⁺ promotes aggregation.

Higher salt concentration also affects the biodiversity in soil [15]. Haj-Amor et al. [24] observed that salt stress alters microbial communities, reduces soil organic carbon (SOC), and affects greenhouse gas (GHG) emissions. These microbial shifts impair soil health, nutrient cycling, and resilience to environmental stresses. Additionally, salinity has an adverse effect on the biological characteristics of soil, including respiration, microbial population, biomass, and enzymatic activities [25]. Sharma et al. [26] investigated how different rice residue particle sizes influenced enzyme activity in normal, saline, and saline-sodic soils. According to the findings, normal soils had the maximum enzymatic activity, followed by saline and sodic soils. Although rice residue incorporation enhanced enzyme activity compared to the control, smaller particle sizes (powdered to 1 cm) proved more effective during a 28-day incubation period. Overall, salinity reduced enzyme effectiveness and residue decomposition, impacting soil biological quality [27].

A reduction in the cation exchange capacity and soil microbial population, as well as an increase in the soil pH, exchangeable sodium percentage (ESP), and sodium adsorption ratio, are only a few of the adverse effects of salinity on soil properties [28]. Saline-sodic conditions are caused by higher accumulation of sodium ions in soil solutions or on cation exchange sites. SAR and ESP values over 13 mmolc kg^−1^ and 15%, respectively, define these soils [29], which severely degrade inherent soil quality and productivity [28].

Salinity significantly reduces soil’s agricultural potential. Water dynamics, salt solubility in the rhizosphere, pH, organic matter content, nutrient availability, structural stability, and redox potential are some of the factors that complicate and control responses at the soil–plant interface under saline field conditions [30]. Salinity reduces soil productivity by impairing nutrient cycling, porosity, carbon fixation, and resistance to biotic stressors. These disruptions contribute to yield losses of 20–50% for many salt-sensitive crops, a situation worsened by climate change [31]. To mitigate salinity stress, long-term strategies such as resource management and crop improvement are essential. On the other hand, simple, affordable biological techniques are also required. Microorganisms that can withstand salty environments generate suitable solutes and encourage plant development by improving tolerance to salinity [32].

**Figure 1:**
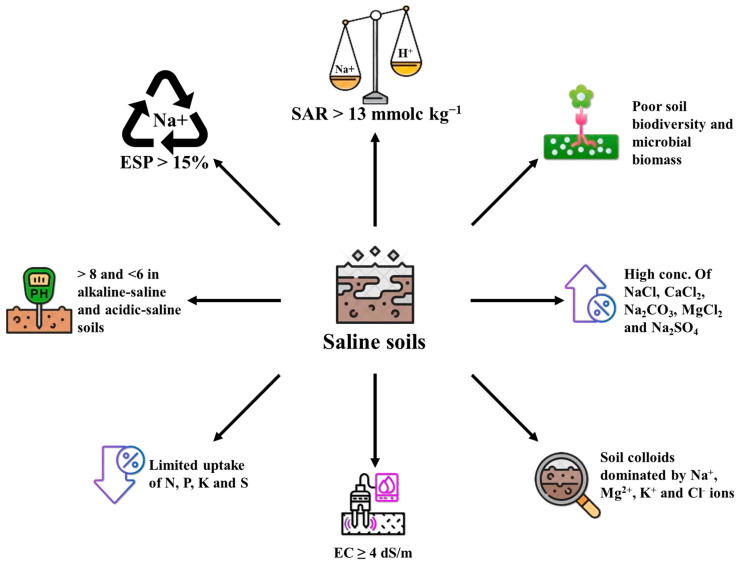
An overview of the properties of saline soils.

The percentage of organic matter, water holding capacity (WHC), water infiltration, soil structure, and stability of aggregates are all decreased by salinity [22]. Elevated salinity and SAR decrease the soil aggregates; stability and soil organic matter. Increased Na^+^ levels increase soil dispersion; however, Ca^2+^ can reverse these effects and encourage aggregate formation [23]. Soil organic matter (SOM) is crucial for water retention. Its contribution to available water-holding capacity (AWHC) varies, though, and is impacted by the mineralogy and texture of the soil. Poor soil structure, high exchangeable sodium levels, decreased permeability, and unbalanced nutrient availability are some of the physical and chemical characteristics of saline soils that have a detrimental impact on plant growth and soil functionality. As shown in Figure 1, saline soils frequently have increased osmotic stress, decreased microbial activity, altered pH, and disrupted nutrient uptake, all of which lower crop productivity and impede sustainable agriculture.

## 3. Effects of Salt Stress on Rice

Salt stress induces multiple physical and physiological changes in *Oryza sativa*, leading to stunted root growth, leaf curling, chlorosis (yellowing), and reduced tillering [33,34]. Additionally, salt-affected rice plants exhibit more empty florets, reduced plant height, smaller grain size, fewer spikelets per panicle, and decreased biomass. Consequently, overall crop productivity declines, with lower grain yield and a reduced harvest index [33]. Salt stress primarily inhibits rice growth through osmotic stress and ionic imbalance, which in turn cause oxidative damage and nutrient deficiencies [35].

### 3.1. Osmotic Stress Mechanism in Rice Under Salinity

Osmotic stress, which is caused by disturbed water flow across cell membranes and elevated osmotic potential, lowers relative water content and chlorophyll levels in rice when it is subjected to salt stress [36]. When external water potential decreases due to salinity, water uptake is restricted, inhibiting root and shoot cell expansion [37,38]. As salinity persists, it further lowers plant cell turgor pressure, reducing cell development [39]. Osmotic stress causes stomatal closure, reducing the plant’s capacity to ingest CO_2_ (owing to a hydraulic signal from roots to shoots) and inhibiting photosynthesis [40]. To lessen the consequences of salt stress, rice plants use osmotic control mechanisms, such as the buildup of osmotic adjustment compounds like proline and soluble carbohydrates [41]. Rice plants store soluble sugars, proline, glycine betaine, and other suitable solutes or osmolytes to deal with osmotic stress. Despite the external osmotic challenge, these organic chemicals assist cells in retaining water and sustaining turgor pressure by lowering the cellular osmotic potential [42].

### 3.2. Mechanism of Ionic Imbalance (Ion Toxicity) in Rice Under Salt Stress

Osmotic stress happens quickly after the plant is exposed to salt stress, whereas ion toxicity from high Na^+^ and Cl^−^ buildup happens later [43]. The accumulation of sodium and chlorine ions in intracellular compartments in rice causes ionic imbalance, also known as ion toxicity, which impairs cellular metabolism and ultimately leads to early leaf drops and plant death [44,45]. There are several pathways that contribute to the toxicity of Na^+^. It initially diminishes the efficiency of enzyme activities, decreases enzyme activity, and negatively impacts metabolism by substituting K^+^ [46]. Additionally, too many sodium ions in the cytoplasm hinder the transport and absorption of K^+^ as well as other macro- and micronutrients, including zinc (Zn^2+^), calcium (Ca^2+^), phosphorus (P), and nitrogen (N) [33]. Na^+^-induced membrane depolarization triggers K^+^ efflux and activates endonucleases and caspase-like proteases, exacerbating cellular damage [47]. Similarly, Cl^−^ toxicity disrupts nutrient absorption, particularly nitrogen and sulfur, further impairing rice performance [48]. Rice plants employ mechanisms such as ion exclusion and tissue tolerance to manage ionic imbalance [49]. It has been shown that low concentrations of NaCl (5 mM) can stimulate plant growth by increasing both shoot and root biomass, in contrast to higher NaCl concentrations that inhibit growth. Under 5 mM NaCl, plants showed increased accumulation of elements like C, S, Zn, and Cu, which were not observed at higher NaCl levels. This enhanced growth was linked to improved photosynthesis and nutrient assimilation, particularly of sulfur, as evidenced by increased cysteine levels. In contrast, high salinity induced stress responses without such beneficial effects, highlighting the potential of low-level NaCl to support plant growth [50].

### 3.3. Mechanism of Oxidative Damage Under Salt Stress in Rice

**Figure 2:**
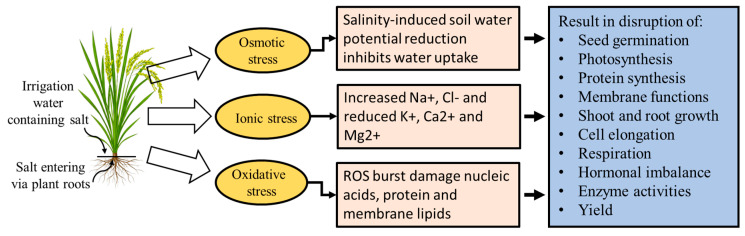
Impact of salt stress on the growth and physiology of plants.

Salinity stress in rice induces osmotic, ionic, and oxidative stress, leading to reduced water uptake, ion imbalance, and ROS-induced cellular damage. These effects disrupt key processes such as germination, photosynthesis, and growth, ultimately lowering yield (Figure 2). The main cause of oxidative damage in rice is the overproduction of reactive oxygen species (ROS), such as H_2_O_2_, superoxide anions, and hydroxyl radicals. ROS accumulates in cells as a result of salt stress, which can cause major damage to cellular structures, lipids, enzymes, and DNA [51]. Salt stress, especially high sodium (Na^+^) accumulation, induces ROS overproduction in rice cells, particularly during sensitive stages like grain filling and seedling growth. This oxidative burst damages organelles and impairs physiological mechanisms [52]. Hasanuzzaman et al. [53] determined that excessive formation of ROS brought on by salinity causes oxidative damage to plant cells, membranes, and organelles, while low concentrations of ROS act as signaling molecules; high concentrations cause oxidative stress, resulting in cell damage and plant death. The elevated level of malondialdehyde (MDA), a byproduct of lipid peroxidation, is a crucial sign of oxidative damage. Increased electrolyte leakage (EL) is frequently observed in conjunction with membrane damage and loss of integrity, which are reflected in elevated MDA levels during salinity [54]. Under salt stress, detoxifying ROS and maintaining ROS balance depend on the antioxidant defense system. Different genes and phytohormones are involved in this defense mechanism. Enhancing the antioxidant system can mitigate salt-induced oxidative damage, helping restore plant growth and function. When exposed to salt stress, rice plants boost their production of both enzymatic and non-enzymatic antioxidants to combat oxidative damage. Key enzymes like superoxide dismutase (SOD), peroxidase (POD), and catalase (CAT) work alongside molecules such as glutathione and ascorbic acid to neutralize ROS and guard cellular structures [55].

### 3.4. Impact of Salt Stress on Rice Quality

When examining how salinity hinders rice—from its growth and metabolism to final yield—we must also consider its impact on grain quality. Factors like nutritional content, cooking properties, and taste can significantly influence both market value and consumer preference. Salt stress significantly impacts rice quality in multiple ways, affecting grain appearance, milling characteristics, nutritional content, and cooking/eating properties. The effects vary depending on the salt concentration, rice variety, and growth stage at which stress occurs. Numerous elements, including cultivating methods, irrigation conditions, climatic change, and variety, affect the quality of rice. Research on rice quality under stressful circumstances is scarce since these elements are harder to regulate in stressful situations [43].

We pay special attention to how salt stress alters the nutritional makeup of rice. In addition to Na^+^ and Cl^−^, saline-alkaline soils are rich in Ca^2+^, Mg^2+^, and a number of trace metals, including Fe^2+^, Mn^2+^, Zn^2+^, and Cu^2+^. Zheng et al. [56] have concluded that salt-stressed rice exhibits a range of nutritional components, including high protein and low starch. Moderate to high levels of salinity during the reproductive stage affect the appearance and milling quality of rice grains. Rice has worse grain appearance and lower milling quality with moderate salinity (~5 dS/m), although its protein content tends to rise [57]. However, at low to moderate salinity levels (below ~17 mM NaCl), salt stress can actually boost milling quality indices, including brown rice rate, milled rice rate, and head milled rice rate, especially in saline-sensitive kinds [58]. Salt stress generally increases the grain protein content by about 12–14% under moderate salinity, which can affect the texture and nutritional value [57]. Starch content and composition are also altered: severe salt stress decreases total starch and amylopectin short-chain content and increases starch crystallinity and gelatinization temperature, leading to poorer cooking and eating quality. Under moderate salt stress, salt-tolerant cultivars may maintain better starch properties, while susceptible cultivars show deterioration [59]. Salinity may have an impact on nutritional quality since it enhances the absorption of sodium, potassium, and magnesium in rice grains while decreasing the absorption of calcium, iron, manganese, and zinc [58].

## 4. Key Sustainable Strategies for Rice Health in Saline Conditions

Salt stress significantly impairs Oryza sativa growth and productivity by osmotic stress, ion imbalance, and oxidative damages, disrupting water uptake, inducing ion toxicity, and causing nutrient imbalance [60,61]. In order to mitigate these impacts, a number of sustainable methods for preserving rice health in saline environments combine genetic, biotechnological, and management techniques. These methods help to increase salt tolerance, stabilize yields, and guarantee long-term productivity in areas affected by salt [17].

### 4.1. Genetic Resources and Breeding Strategies

#### 4.1.1. Utilization of Wild Relatives and Novel Genetic Resources

*Oryza rufipogon* and *Oryza nivara* are two examples of wild rice species that are useful genetic resources for improving farmed rice’s resistance to salt. These wild species have been utilized to create enhanced rice varieties, which show greater salt tolerance since they are naturally suited to saline settings and feature mechanisms like efficient Na^+^ exclusion, K^+^ retention, and osmotic adjustment [62,63]. Extremely diverse genetic loci linked to distinct adaptive qualities are present in wild progenitors that have evolved to a variety of shifting environmental circumstances [64]. Wild rice species have been used to introgress salt tolerance traits into cultivated rice. Elite introgression lines derived from crosses with wild relatives exhibit improved root traits, ion regulation, and overall salt tolerance [62]. Next-generation sequencing (NGS) and genome-wide association studies (GWAS) technologies help identify new alleles from wild germplasm, resolving cross-compatibility issues and improving the genetic foundation for salt tolerance [62]. The creation of salt-tolerant cultivars can be aided by taking advantage of the genetic variety of rice germplasm from gene banks [65].

#### 4.1.2. Marker-Assisted Selection and Speed-Breeding

Together with speed-breeding techniques, Marker-Assisted Selection (MAS) is a powerful method to accelerate the development of salt-tolerant rice cultivars by enabling precise gene introgression and rapid generation progress. SNP Marker-Assisted Selection and speed-breeding are two techniques used to create salt-tolerant rice cultivars. Using these techniques, for example, the *hst1* gene was introduced into high-yielding rice cultivars, enhancing their resistance to salinity [66,67]. MAS effectively transfers salt tolerance characteristics into high-yielding but salt-sensitive cultivars by using genetic markers connected to salt tolerance genes or QTLs, such as the well-known Saltol locus from the tolerant parent FL478. For example, Saltol was introgressed into the popular variety ADT 45 using MAS, resulting in new lines with improved seedling-stage salinity tolerance and good agronomic performance [68]. MAS allows early and accurate selection of plants carrying desired alleles, reducing linkage drag by restricting donor genome segments and speeding up breeding cycles compared to conventional methods. Markers like RM3412, AP3206, RM8094, and RM493 are commonly used to track Saltol during breeding [68]. Speed-breeding accelerates rice breeding by manipulating environmental conditions (e.g., temperature, photoperiod, spacing, nutrient management) to shorten generation time, enabling up to five generations per year instead of 1–2 under traditional field conditions [69].

#### 4.1.3. Transgenic Approaches

**Figure 3:**
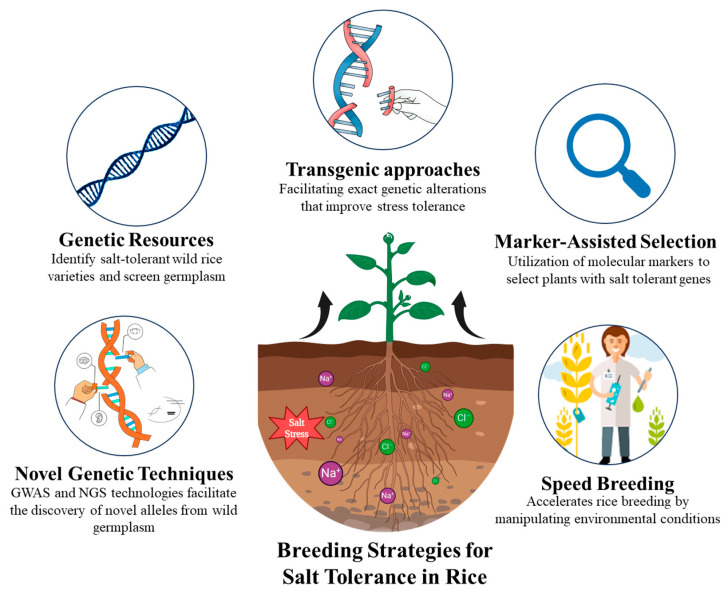
Summary of breeding strategies for enhancing salt tolerance in rice.

By enabling precise genetic changes that increase stress tolerance, transgenic techniques have significantly advanced the development of rice cultivars resistant to salt. To help rice thrive in salty soils, scientists are turning to genetic strategies that improve ion equilibrium, balance water uptake, and combat oxidative stress. For example, boosting the activity of key genes like *OsHKT1;5* and *OsNHX1* helps rice plants exclude excess sodium and safely store it away—keeping the critical sodium–potassium balance in check even under saline conditions [70]. Researchers have also developed genetically modified rice varieties, like those carrying the *SaPMP3* gene from coastal cordgrass, which show better salt tolerance by maintaining proper ion balance and improving survival in salty soils [71]. Scientists are boosting rice’s salt tolerance by borrowing helpful genes from salt-loving plants. For example, the *SaVHAc1* gene from cordgrass helps rice better regulate its cellular processes, making it more resilient in salty conditions. Under high salinity conditions, transgenic rice, such as *SaVHAc1* and *SaPMP3*, shows higher grain yield, reduced ion toxicity, and increased chlorophyll retention [72]. Additionally, RNA interference-mediated suppression of stress-sensitive genes, particularly *OsDSR2* silencing, increases antioxidant activity, reduces oxidative damage, and increases proline formation, all of which lead to higher survival rates. [73]. The various strategies employed by researchers to mitigate salinity issues are visually summarized in Figure 3.

Rice adjusts to salinity through complex physiological and biochemical mechanisms, including the regulation of genes that respond to stress (Table 1). A key survival strategy involves maintaining cellular ion homeostasis by tightly controlling ion uptake, sequestering excess Na^+^ and Cl^−^ into vacuoles, and actively excluding toxic ions from sensitive tissues [74].

### 4.2. Management Strategies for Salt Tolerance in Rice

#### 4.2.1. Agronomic Approaches

**Figure 4:**
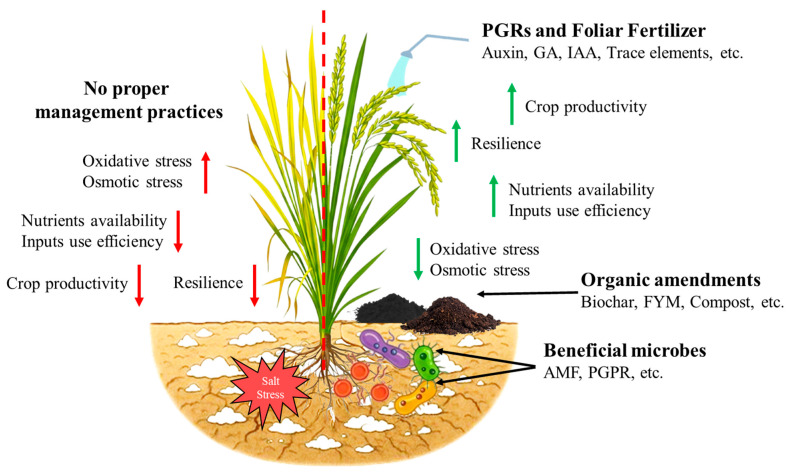
Overview of management strategies for salt tolerance in rice. The summary of management strategies, including the effect of agronomic approaches, organic amendments and fertilization, symbiotic microbes, and plant growth regulators for salt tolerance in rice. Upward arrows mean an increase, and downward arrows mean a decrease. Green arrows show a positive change, while red arrows show a negative change.

Developing and selecting salt-tolerant rice varieties is crucial for improving productivity in saline environments. Varieties like CSR43 and CSR30 have been identified for their high yield and disease resistance in sodic soils, showing better performance compared to local varieties [124,125]. Proper agronomic techniques, such as optimized nutrient management and planting techniques, are essential for enhancing rice productivity in saline soils. For instance, transplanting four seedlings per hill at specific spacing and using an optimal nitrogen application rate can significantly increase yields [125]. Furthermore, combining location-specific agronomic techniques with salt-tolerant cultivars can increase resilience and close yield gaps in salt-affected regions. The concentration of salt in the rhizosphere can be decreased by utilizing appropriate irrigation techniques, such as flushing saline soils with fresh water, keeping a shallow water layer while rice is growing, and scheduling watering to prevent salt accumulation [68]. Integrating crop rotation with salt-tolerant rice varieties helps manage soil salinity and break pest and disease cycles [126]. Crop residues and appropriate tillage improve soil organic matter and water retention, contributing to better salt stress resilience [126]. Additionally, agronomic interventions, including the use of AMF, organic amendments, and micronutrient supplementation, have shown promise in enhancing rice resilience under saline conditions (Figure 4). These integrated approaches offer sustainable solutions to mitigate salt stress, ensuring better crop performance in saline-affected soils.

#### 4.2.2. Organic Amendments and Fertilization

The application of ameliorants like gypsum and gypsum phosphate improves soil structure and reduces sodium toxicity. Balanced mineral nutrition, especially adequate nitrogen and potassium supply, supports salt tolerance by enhancing growth and metabolic functions. Organic amendments also improve soil health and microbial activity, indirectly mitigating salt stress. Recently, the application of different organic matter supplements has shown promise as a means of encouraging plant growth in salt-stressed environments. Numerous studies have shown that these amendments greatly decrease oxidative and osmotic stress in plants by increasing microbial activity [127]. By adding carbon-containing, energy-rich substances through organic amendments, soil microbial populations are able to produce osmolytes, which reduce the osmotic pressure brought on by excessive salinity [27]. Organic amendments, particularly when combined with compost, have shown considerable benefits in improving saline soil ecosystems [128]. It has been shown that adding organic matter to salty soils speeds up the dissolution of calcite (CaCO_3_) by encouraging the quick production of carbonic acid. Smaller particles can more easily bond to the soil as a result of this process, creating stable aggregates that can withstand wet conditions. The potential of several organic soil additives, including hydrochar, biochar, farmyard manure, chicken manure, and manure composites, to improve the chemical and physical characteristics of saline-alkaline soils has been thoroughly investigated. It has been demonstrated that these amendments decrease soil pH, lower salt levels, and lessen stress brought on by salinity [129]. Farmyard manure (FYM) is a nutrient-rich compost made from cow dung, urine, bedding, and other dairy waste. Packed with nitrogen, phosphorus, and essential trace minerals, it not only enriches soil fertility but also improves its overall structure through stable organic matter [130]. In saline-sodic soils, it efficiently lowers pH and EC while supporting the activities of soil flora and fauna as a vital source of soil C.

Organic amendments have also been reported to significantly improve root fresh weight under salt stress, with increases of 48%, 39%, and 84% observed in maize plants [131]. These natural amendments do not just help plants thrive in salty soils but also support long-term farming sustainability. Yang et al. [132] determined that applying organic amendments has been thought to be a successful strategy for reversing the soil deterioration caused by salt. Their findings demonstrated that adding organic matter to salty soil might raise soil organic matter and lower pH, both of which could directly encourage plant development. By improving nutrient cycling, the application of organic amendments increased soil enzymatic activity, which may indirectly encourage plant growth.

#### 4.2.3. Symbiotic Microbes Enhancing Salt Tolerance in Rice

By facilitating nutrient intake, controlling ion balance, reducing oxidative stress, and encouraging development in saline environments, symbiotic microorganisms are essential for increasing rice’s salt tolerance. The main players include salt-tolerant plant growth-promoting rhizobacteria (PGPR), endophytic bacteria, and arbuscular mycorrhizal fungi (AMF).

Many rhizospheric bacteria isolated from saline rice fields exhibit a range of traits that support plant growth, such as siderophore production, phosphate and zinc solubilization, ammonia synthesis, and indole-3-acetic acid (IAA) via both tryptophan-dependent and independent pathways. These bacteria can tolerate extremely high salt concentrations. Under salt stress, these characteristics promote rice growth and improve nutrient availability [133]. PGPR enhance salinity tolerance via phytohormone production, ACC deaminase activity, and exopolysaccharide secretion [134,135]. Hormones, including auxin, cytokinin, and gibberellin, are induced by PGPR, but ACC deaminase can suppress hormones like ethylene. While ethylene plays a part in plant growth and development, high levels of ethylene can be detrimental and stop plants from developing. PGPR help plants better tolerate salt stress by regulating ethylene levels through their ACC deaminase activity [136]. PGPR also help plants combat salt stress by boosting their antioxidant defenses, balancing ion levels, and activating stress-responsive genes [135]. For instance, it has been shown that *Klebsiella* sp. SBP-8 increases K^+^ absorption (84.21%) and Na^+^ exclusion (65%) to improve wheat development under saline conditions [137]. SBP-8 increased ACC deaminase activity by 6%, confirming its plant-growth-promoting role in saline environments [137]. Therefore, in saline conditions, bacteria that have ACCD and are salt tolerant may be advantageous, benefiting plants. Furthermore, it has been shown that PGPR improves phosphate solubilization, biological nitrogen fixation, and nutrient utilization [138]. An affordable and sustainable method of increasing crop yield in salty environments is to use PGPR as a biological agent to lessen salt stress [134,138].

Endophytic bacteria isolated from halotolerant plants, like *Curtobacterium oceanosedimentum*, *Enterobacter ludwigii*, and *Bacillus cereus*, significantly improve rice growth under salt stress by producing phytohormones (IAA, gibberellins), organic acids, and enhancing antioxidant capacity. These endophytes reduce endogenous abscisic acid (ABA) levels in rice under salt stress while increasing glutathione (GSH) and sugar contents, which contribute to osmoprotection and ROS scavenging. Additionally, they increase the expression of genes (*OsYUCCA1* and *OsPIN1*) involved in auxin production and transport, which promotes root and shoot development [139].

By improving soil structure, boosting water and nutrient absorption, and controlling physiological and biochemical processes inside the plant, AMF establish symbiotic relationships with rice roots and increase salt tolerance. Arbuscular mycorrhizal fungi invade plant roots, fostering development and enhancing the plant’s natural defenses against salt stress. These results have shown that AMF’s alleviation of saline soil stress conditions is linked to improvements in photosynthetic rate, osmoregulator accumulation, nutrient absorption, and water usage efficiency [140,141].

AMF symbiosis has also been proposed to alleviate salt stress in plant hosts in a number of ways. These include biochemical, physiological, molecular, and ultrastructural alterations, plant growth, and biomass [142]. Saline conditions impede plant development and biomass allocation. This might be a result of the increased osmotic potential of the salt-affected soils, which prevents plants from absorbing nutrients. Nonetheless, it has been shown that AMF root colonization improves growth and biomass allocation by increasing the host plant’s nutrient intake. AMF-inoculated seedlings showed higher shoot and root dry masses than uninoculated mycorrhizal seedlings [143]. In comparison to control plants, a mycorrhizal tomato plant also showed higher fresh fruit output, fruit mass, number of fruits, and shoot and root dry mass [144]. The enhanced nutrient intake facilitated by AMF, namely enhanced phosphorous nutrition and improved plant development, is associated with AMF symbiosis [145]. Collectively, these results demonstrated how symbiosis helps agricultural plant cultivars cope with saline stress.

#### 4.2.4. Plant Growth Regulators (PGRs)

Plants naturally biosynthesize PGRs, which alter crop plant development (increased branching and rebranching, shoot and root growth, reproduction, etc.) and are important in reducing abiotic stressors [146]. By modifying physiological, biochemical, and molecular reactions that lessen the negative effects of salinity stress, PGRs significantly contribute to the improvement of rice’s salt tolerance. By increasing non-enzymatic antioxidants (ascorbate and glutathione), decreasing ROS accumulation, lowering malondialdehyde (MDA) content, and enhancing antioxidant enzyme activities (e.g., peroxidase, catalase, and ascorbate peroxidase), foliar application of PGRs such as 5-aminolevulinic acid (5-ALA) and DTA-6 protects rice seedlings from oxidative damage [54]. The application of PGRs increases beneficial hormones such as gibberellic acid (GA3), jasmonic acid (JA), indole-3-acetic acid (IAA), salicylic acid (SA), and zeatin riboside (ZR) while reducing abscisic acid (ABA) content in roots. This hormonal modulation improves root morphology, root vigor, and overall plant growth under salinity [147]. Growth regulators like naphthalene acetic acid (NAA), kinetin (KIN), gibberellic acid (GA), and brassinolide (BR) have been shown to mitigate salinity-induced reductions in plant growth and development. Abscisic acid (ABA) administration frequently decreased growth metrics, but NAA application dramatically boosted plant height and productivity under saline irrigation as compared to control [148]. PGRs like gibberellic acid and BR increase the rate of photosynthetic activity and chlorophyll fluorescence under salt stress, increasing the plant’s ability to continue photosynthesis in spite of unfavorable circumstances [148].

### 4.3. Primary and Secondary Metabolites in Salt-Stressed Rice

In saline environments, rice relies on both primary and secondary metabolites to maintain physiological functions and enhance stress tolerance. Primary metabolites, including sugars like sucrose and glucose, amino acids such as proline, and organic acids including citrate and malate, play essential roles as osmoprotectants and detoxifiers [49,149]. These compounds help balance cytoplasmic water potential, stabilize enzymatic activities, and scavenge reactive oxygen species (ROS), with salt-tolerant rice varieties typically accumulating higher concentrations of these metabolites compared to sensitive cultivars [150,151]. For instance, proline serves dual functions as both an osmolyte and antioxidant, while organic acids participate in pH regulation and ion homeostasis, mirroring metabolic responses observed in halophytic species [152,153].

**Figure 5:**
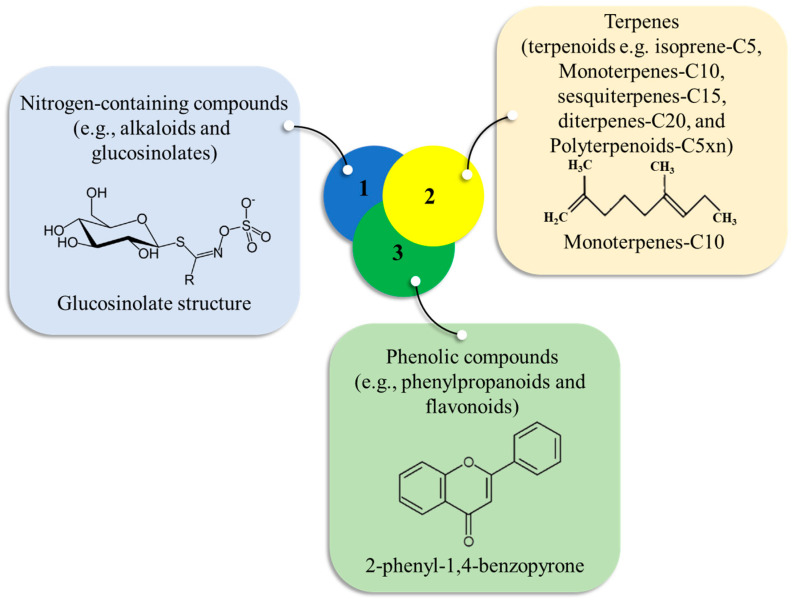
Diagrammatic illustration of the secondary metabolite classes found in rice.

In addition to primary metabolites, rice activates diverse secondary metabolic pathways to combat salinity stress. These secondary compounds, which include phenolic compounds like flavonoids and lignins, nitrogen-containing polyamines, and terpenoids such as momilactones (Figure 5), function as antioxidants, structural reinforcements, and signaling molecules [154,155]. Phenolic compounds, particularly flavonoids, protect photosynthetic apparatuses from ROS damage, while lignins fortify cell walls to restrict sodium influx [46]. The nitrogen-containing polyamines stabilize nucleic acids and membranes, and terpenoid phytoalexins like momilactones contribute to both defense and osmotic adjustment [156]. Studies have shown that salt-tolerant rice varieties upregulate these secondary metabolites more effectively than sensitive ones, with increased accumulation of anthocyanins, jasmonic acid, and triterpenoids in salt stress [157].

The coordinated action of primary and secondary metabolites in rice not only mitigates immediate salt stress effects but also supports long-term adaptation. This metabolic flexibility highlights potential targets for breeding and biotechnological interventions, such as engineering rice lines with enhanced osmolyte production or applying exogenous metabolites to boost stress resilience [157]. Understanding these metabolic responses is crucial for developing rice genotypes capable of sustaining productivity in saline environments, thereby addressing challenges posed by soil salinization in agriculture.

### 4.4. Modification of Plant Antioxidant Pathways in Salt-Stressed Rice

**Figure 6:**
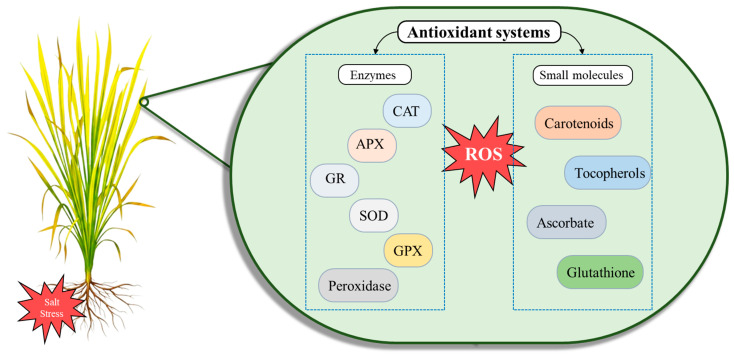
Antioxidant components in plants defend against ROS brought on by salt stress.

To combat salt-induced oxidative damage, *Oryza sativa* employs a robust antioxidant defense system that consists of both enzymatic and non-enzymatic elements. In addition to defensive enzymes like SOD, CAT, APX, and GPX, the system depends on vital antioxidants such as ascorbate, glutathione, carotenoids, and tocopherols (Figure 6) [158]. This defense depends on the ascorbate–glutathione cycle, and enzymes such as glutathione reductase (GR) and APX are essential for salt tolerance [159].

Rice plants suffer oxidative damage in saline conditions, making antioxidant capability critical for survival. Salt-tolerant rice varieties frequently exhibit higher amounts of antioxidants, including ascorbate and protective enzymes like SOD, peroxidase (POD), and APX, which work together to neutralize reactive oxygen species (ROS) [160,161]. These cultivars also show reduced malondialdehyde (MDA) levels, indicating less membrane lipid peroxidation and better cellular integrity under saline conditions [162,163]. Rice has evolved specific genetic mechanisms to bolster its antioxidant defense. Key genes involved in salt tolerance include *OsSOD1* and *OsSOD2* (superoxide dismutase genes) that enhance ROS scavenging, *OsAPX2* plays a crucial role in H_2_O_2_ detoxification, *OsGR1* (glutathione reductase) is essential for maintaining the reduced glutathione pool, *OsCAT* (catalase) genes that break down hydrogen peroxide, and *OsPRX* (peroxidase) genes are involved in lignin formation and ROS elimination. Studies demonstrate that overexpression of these antioxidant-related genes can significantly improve salt tolerance in rice. For instance, upregulation of *OsAPX2* has been shown to enhance salinity tolerance by maintaining redox homeostasis [126]. Similarly, *OsSOD1* overexpression reduces oxidative damage in salt-stressed rice seedlings [54].

The understanding of rice’s antioxidant regulation has important implications for breeding programs. Genetic engineering approaches targeting these antioxidant genes offer promising strategies to develop salt-tolerant cultivars. CRISPR/Cas9-mediated editing of *OsGR1* has shown potential in enhancing glutathione recycling under stress [126]. Furthermore, manipulating the expression of transcription factors like *OsDREB1A* that regulate multiple antioxidant genes could provide broad-spectrum stress tolerance [164]. This comprehensive antioxidant defense system, coupled with genetic insights into rice-specific tolerance mechanisms, provides valuable targets for improving rice productivity in salinity. Future research should focus on elucidating the complex regulatory networks controlling these antioxidant pathways to develop more resilient rice cultivars.

## 5. Conclusions and Future Perspectives

Rice faces multifaceted challenges under salinity stress, necessitating holistic approaches for sustainable crop production. This review outlines how integrating metabolic regulation, microbial partnerships, organic inputs, and hormonal modulation can significantly enhance rice performance in saline soils. The application of gene-editing tools, combined with traditional breeding and microbial-assisted strategies, shows tremendous promise in engineering salt-tolerant rice cultivars. Importantly, advances in metabolomics, transcriptomics, and epigenomics are accelerating the understanding of rice adaptation mechanisms at the cellular and molecular levels. However, direct application in practice necessitates further experimental validation, typically involving the cloning and introduction of these specific genes into host organisms to confirm their functional impact. A collaborative effort between scientists, breeders, and policymakers is essential to translate these innovations into resilient agricultural practices for saline-prone regions. While extensive research has already focused on developing and field-testing salt-tolerant rice genotypes using microbial and biotechnological methods, a persistent challenge remains in achieving stable and durable salt tolerance under varying field conditions. This susceptibility can be attributed to complex environmental dynamics, as well as biological and microbial shifts.

Consequently, research continues to focus on identifying novel target genes capable of conferring more effective and robust salt tolerance to rice. Leveraging multi-omics platforms can elucidate complex stress-responsive networks, enabling precision breeding and metabolic engineering. Research should also optimize the formulation and delivery of microbial consortia and organic amendments tailored to specific soil types and stress levels. Long-term monitoring of soil health and crop productivity under such interventions will inform sustainable practices. Additionally, integrating farmer-centered approaches and local knowledge can enhance the adoption of salt-resilient technologies. Addressing soil salinization in rice-growing areas is not just a scientific challenge but a socio-economic necessity to ensure food security under climate change.

## Figures and Tables

**Table 1:** List of stress-responsive genes leads to salinity stress tolerance in rice.

Gene Name	Gene Function	Changes in Mutants/Validation	Reference
*OsBGE3*	Cytokinin transport	Reduced grain length; salt hypersensitivity	[75]
*OsDST*	Zinc finger TF	Broad leaves; decreased stomatal density; improved salt tolerance	[76]
*OsFLN2*	Sucrose metabolism	Salt sensitivity due to inadequate assimilated supply	[77]
*OsGTy-2*	Trihelix TF	Increased root Na^+^/K^+^ and high Na^+^/K^+^ ratio; salt hypersensitivity.	[78]
*OsPIL14*	Basic helix-loop-helix TF	Reduced coleoptile and root elongation	[79]
*OsPQR3*	E3 ubiquitin ligase	Enhanced oxidative/salt tolerance; upregulated *OsGPX1*, *OsAPX1*, *OsSOD1*	[80]
*OsRR9*, *OsRR10*	Cytokinin signaling	High salt tolerance; upregulated ion transporters	[81]
*OsDOF15*	Transcription factor	Short roots; impaired meristem activity; salt hypersensitivity	[82]
*OsSPL10*	Transcription factor	Glabrous leaves; enhanced seedling survival under salt	[83]
*OsOTS1*	SUMOylation	Reduced chlorophyll/root biomass; salt sensitivity	[84]
*OsRR22*	Cytokinin signaling TF	Increased shoot biomass; improved salt tolerance	[85]
*OsNCA1a*, *OsNCA1b*	–	Cell death; salt sensitivity	[86]
*OsNAC041*	–	Reduced germination; high ROS/MDA; salt sensitivity	[87]
*OsBBS1*	Receptor-like kinase	Early senescence; reduced root length/tillers; salt hypersensitivity	[88]
*OsMIR528*	miRNA	Delayed branching; chlorosis	[89]
*OsRAV2*	Brassinosteroid-response TF	Loss of salt-induced expression	[90]
*OsHAK3*	Potassium transporter involved in K^+^/Na^+^ balance	Candidate gene for salt tolerance at germination; mutants show salt sensitivity; important for ionic homeostasis	[91]
*OsITPK5*	Inositol trisphosphate kinase involved in stress signaling	Identified as candidate gene for salt tolerance at germination stage; likely role in signaling and adaptation	[91]
*OsWRKY53*	Transcription factor regulating salt response gene	Binds promoters of *OsHKT1;5* and *OsMKK10.2*; elite haplotypes associated with improved salt tolerance	[92]
*SKC1*/*OsHKT1;5*	Sodium transporter maintaining Na^+^ exclusion from shoots	Salt tolerance locus; expression positively correlated with *OsWRKY53*; key for ionic homeostasis	[92]
*miR396b*/*GRF6 module*	Regulatory module controlling salt stress response via MYB3R TF	Enhances salt tolerance by increasing ROS scavenging enzymes; MYB3R is direct target; overexpression improves survival	[93]
*OsFLP*	R2R3 MYB-like TF regulating stomatal development	Identified as salt tolerance candidate via trans-eQTL analysis; involved in adaptive response to salinity	[94]
*OsCYP2*	Cyclophilin protein confers salt tolerance	Identified as beneficial under saline conditions; involved in protein folding and stress response	[94]
*DMS3/OsITPK2*	Stress signaling and chromatin remodeling	Critical in salt tolerance; related to inositol phosphate metabolism	[91]
*OsbHLH024*	Negative regulator of salt stress; affects ion balance and antioxidant activity	Knockout mutants exhibited improved salt resistance and upregulation of ion transporter genes	[95]
*OsRR22*	Main effect gene for salt tolerance; loss-of-function increases tolerance	CRISPR/Cas9 generates knockout lines with enhanced salt tolerance	[96]
*OsSPL10*	Influences rhizosphere microbiota and ion accumulation under salt stress	CRISPR/Cas9-edited lines with loss of function showed better adaptation to salt stress	[97]
*OsDSG1*	Involved in ubiquitination pathway; regulates biochemical reactions under salt stress	CRISPR/Cas9-induced mutants displayed enhanced salt tolerance at germination and seedling stages	[98]
*OsSHMT3*	Photorespiration	Overexpression	[99]
*SIDP361*	Proline	Overexpression	[100]
*OsNHX1*	Compartmentalization of Na+ into vacuoles	Overexpression	[101]
*OsCIPK15*	Enhanced salt tolerance	Overexpression	[102]
*CYP94C2b*	Deactivation of jasmonate	Overexpression	[103]
*Oshkt1;1*	Sensitive to salt stress	Mutant studies	[104]
*OsHKT2:1*	Na^+^ accumulation under low K^+^ supply	Overexpression	[105]
*OsHAK5*	Root K acquisition and transport to shoot at low K levels	Overexpression	[106]
*OsSOS1*	Improved salt tolerance	Transformation	[107]
*SAPK4*	Improved salt tolerance	Transgenic	[108]
*AKT1*	Enhances K^+^ uptake	Overexpression	[109]
*OsHAK21*	Na^+^/K^+^ homeostasis	Quantitative expression	[110]
*OsPP1a*	Enhanced tolerance to high salinity/upregulation of SOD	Transgenic	[111]
*PtCYP714A3*	Shoot response to salt toxicity	Transgenics (ectopic expression)	[112]
*OsSUV3*	Salinity tolerance by maintaining photosynthesis and antioxidant machinery	Transgenic	[113]
*OsRMC*	Negative regulation of salt tolerance	Knock down expression	[114]
*OsCPK4*	Enhances salt and drought tolerance	Overexpression	[115]
*P5CS*	High accumulation of proline	Transgenic	[116]
*codA*	Promotes synthesis of glycine betaine	Transgenic	[117]
*OsTPS1*	Enhances salt tolerance	Overexpression	[118]
*PINO1*	Allows growth of transgenic plants in salt environment	Introgression and expression	[119]
*OsTIP1;1*	Upregulation in salt stress	Overexpression	[120]
*HvPIP2;1*	Enhances sensitivity to salinity	Overexpression	[121]
*OsDREB1A*, *OsDREB1F*, *OsDREB2A*	Improved salt tolerance	Transgenic	[122]
*OsCDPK7*	Enhances salt tolerance	Transgenic	[123]
